# Systematic analysis of the relationship between ovarian cancer prognosis and alternative splicing

**DOI:** 10.1186/s13048-021-00866-1

**Published:** 2021-09-15

**Authors:** Di Zhang, Dan Zou, Yue Deng, Lihua Yang

**Affiliations:** Department of Gynaecology, the 2nd Afliated Hospital of Kunming Medical University, Kunming, Yunnan China

**Keywords:** Ovarian cancer, Alternative splicing, Survival model, Prognosis, Splicing factors

## Abstract

**Background:**

Ovarian cancer(OC) is the gynecological tumor with the highest mortality rate, effective biomarkers are of great significance in improving its prognosis. In recent years, there have been many studies on alternative splicing (AS) events, and the role of AS events in tumor has become a focus of attention.

**Methods:**

Data were downloaded from the TCGA database and Univariate Cox regression analysis was performed to determine AS events associated with OC prognosis.Eight prognostic models of OC were constructed in R package, and the accuracy of the models were evaluated by the time-dependent receiver operating characteristic (ROC) curves.Eight types of survival curves were drawn to evaluate the differences between the high and low risk groups.Independent prognostic factors of OC were analyzed by single factor independent analysis and multi-factor independent prognostic analysis.Again, Univariate Cox regression analysis was used to analyze the relationship between splicing factors(SF) and AS events, and Gene Ontology(GO) and Kyoto Encyclopedia of Genes and Genomes(KEGG) enrichment analysis were performed on OS-related SFs to understand the pathways.

**Results:**

Univariate Cox regression analysis showed that among the 15,278 genes, there were 31,286 overall survival (OS) related AS events, among which 1524 AS events were significantly correlated with OS. The area under the time-dependent receiver operating characteristic curve (AUC) of AT and ME were the largest and the RI was the smallest,which were 0.757 and 0.68 respectively. The constructed models have good value for the prognosis assessment of OC patients. Among the eight survival curves, AP was the most significant difference between the high and low risk groups, with a P value of 1.61e − 1.The results of single factor independent analysis and multi-factor independent prognostic analysis showed that risk score calculated by the model and age could be used as independent risk factors.According to univariate COX regression analysis,109 SFs were correlated with AS events and adjusted in two ways: positive and negative.

**Conclusions:**

SFs and AS events can directly or indirectly affect the prognosis of OC patients. It is very important to find effective prognostic markers to improve the survival rate of OC.

**Supplementary Information:**

The online version contains supplementary material available at 10.1186/s13048-021-00866-1.

## Introduction

For humans, all the important components of the body need proteins, and the diversity of proteins provides the basis for the diversity of body functions. AS as an important mechanism for post-transcriptional regulation of gene expression, which can produce the same, similar or even opposite proteins and provide a basis for the expansion of functional proteome diversity in eukaryotic genes when acts on precursor messenger RNA (pre-mRNA) [1]. AS events occur between 92% and 94% of human genes, a higher proportion than many other species and exactly in line with the fact that humans have more complex morphology and behavior [2]. Many studies have shown that AS events related to the initiation and progression of many diseases, from the initial research in the Mediterranean anemia and spinal muscular atrophy , in recent years glioblastoma, clear cell carcinoma, renal cell carcinoma and hepatocellular carcinoma (HCC) and so on many kinds of cancer are discussed [3–6]. More and more studies have shown that AS can affect the progress of cancer through multiple biological processes such AS cell proliferation , apoptosis, tumor invasion and metastasis [7–9]. Studies have reported that AS events occur more frequently in cancer tissues than in normal tissues, and changes can occur in both exons and introns [10–12]. SF genes act as transcription factors to recognize the cis-regulatory element in pre-mRNA, then promote exon selection and splicing site selection [13]. SFs play important role in tumors by affect AS events in a variety of ways [14].

OC is the most fatal disease among gynecological malignancies. It is the 10th leading cause of cancer death among women in China and the fifth leading cause of cancer death among women globally. According to statistics, the five-year survival rate of 2010-2016 in OC was 48.6%(https://seer.cancer.gov/statfacts/html/ovary.html) .In 2018, there were 295,414 OC patients in the world, among which 52,971 were in China. The late diagnosis and the occurrence of platinum-resistance have great impact on the prognosis of patients. It is important to discover high sensitive and specific biomarkers for OC patients. Previous studies have shown that there are close association between AS events and OC, but the systematic analysis of them on the prognosis is still not comprehensive [15]. Similarly, the relationship between splicing factor and OC is less about prognosis.

Bioinformatics technology can use biological algorithms and related software tools to collect, process, store, analyze and interpret biological data. High-throughput sequencing technology is a relatively mature sequencing technology in recent years, which makes it possible to analyze the whole transcriptome and genome of species. In the study of AS events, RNA sequencing technology(RNA-seq) of high-throughput sequencing technology was mostly adopted, and full transcriptome sequencing was performed at the transcriptome level. Studies have shown that RNA-seq is also very good at detecting those genes closely related to OC [16, 17]. In this study, a large number of OC raw data were obtained from the TCGA database, and a systematic analysis of AS events and the prognosis of OC patients was conducted from aspects such AS image and signal processing by using bioinformatics software combined with RNA-seq. We found that a large number of AS events were related to the OS of OC and SFs, and the constructed prognostic models were of good value in evaluating the prognosis of patients.

## Material and methods

### Data acquisition and preprocessing

The clinical and transcriptomic data of 587 OC samples were downloaded from TCGA database (https://tcga-data.nci.nih.gov/tcga/). The sample ID was converted to the form of "OSCAR|51769|AA" (OSCAR is gene symbol, 51769 is ID number and AA is splicing type), samples with unquantifiable values greater than 30% were deleted. The Percent- splice-in (PSI) value of AS events were download from the TCGA SpliceSeq database (https://bioinformatics.mdanderson.org/TCGASpliceSeq/PSIdownload.jsp), which included seven types AS events with PSI values: Exon skip (ES), both intron (RI), Alternate Donor site (AD), Alternate acceptor site (AA), Alternate promoter (AP), Alternate terminator (AT), and Channel exclusive exons (ME). The PSI values ranged from 0% to 100%, the data with mean PSI <0.05, fluctuation <0.01, NA probability >30% and unknown survival state were deleted . Finally, 384 samples with survival time more than 90 days were included in the study. Through other articles [18], we obtained 404 human SF genes for this study.

### Getting the survival-associated AS events

Univariate Cox regression analysis was used to obtain OS-related AS events in R4.0.3. The relationship between AS events and prognosis of OC was understood by drawing volcano plot. Volcano Plot based on the correspondence between -log10 and Z value, red represents the AS events with *P*<0.05, and blue represents the AS events with *P*>0.05. The comparison of seven types AS events were made in Upset plot. Histogram shows the high and low risk group in each type according to the relationship between HR value and the prognosis.In the bubble diagram, the correlation between AS events and prognosis was indicated by color and circular size.

### Building and evaluating survival model

The optimal number of AS events were obtained by Least absolute shrinkage and selection operator (LASSO) regression. The screened AS events were analyzed by R package and the survival model formula was constructed as follows:$Riskscore = \sum\nolimits_{i}^{n} {PSI\beta {\text{i}}}$ (βis the regression coefficien_t_) [5]. After calculating the median value of the risk score, we divided the samples into high risk group and low risk group. Kaplan–Meier survival curves were drawn and the difference of the two groups were represented by the *P* value [19]. In addition, the relationship between the risk score and prognosis in OC patients were analyzed by risk score curves, survival status diagrams and PSI value heatmap jointly. The accuracy of survival models was evaluated by ROC. Then, single and multi-factor independent prognostic analysis were carried out to evaluate the independent factors related to the prognosis of OC patients.

### Constructing correlation network between SF genes and AS events

The date that absolute values of correlation coefficient >0.4 (Z value) and *P* value <0.001 were screened, then the network map between SF genes and AS events was constructed in Cytoscape to demonstrate the regulatory role of SF gene and AS events in OC. Joint use of DAVID database (https://david.ncifcrf.gov/) and KOBAS database (http://kobas.cbi.pku.edu.cn/kobas3) to Gene Ontology(GO) and Kyoto Encyclopedia of Genes and Genomes(KEGG) analysis for the 109 SFs.Similarly, R was used to visualize the KEGG analysis results and the top 20 GO analysis pathways with the smallest *P* values.

## Results

### Different types of AS events in OC

**Fig. 1:**
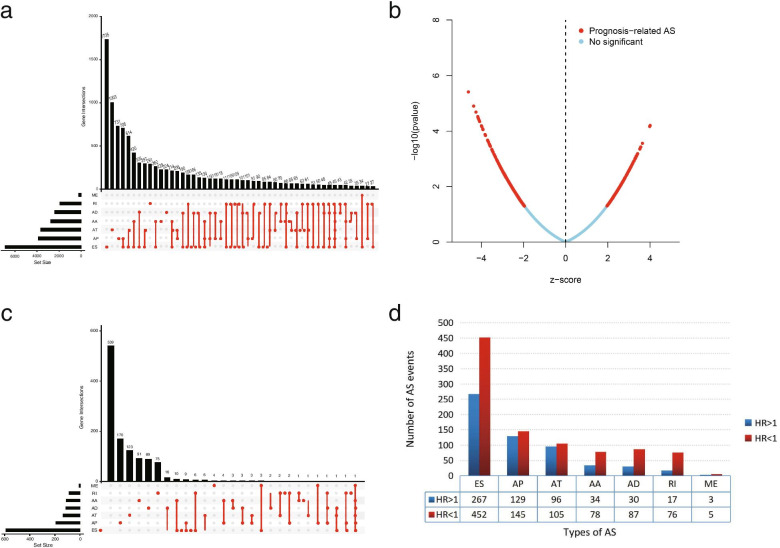
Total analysis of alternative splicing events and ovarian cancer. **a**, An Upset plot displaying the intersections of different types of alternative splicing events associated with survival in ovarian cancer in ovarian cancer. **b**, Volcanic Plot showing the strength of association between alternative splicing events and ovarian cancer. **c**, An Upset plot displaying the intersections of different types of alternative splicing events significantly associated with survival in ovarian cancer in ovarian cancer (*P* < 0.05). **d**, Among alternative splicing events significantly associated with ovarian cancer survival, the number of positive (HR < 1) or negative (HR > 1) events. AA, alternate acceptor site; AD, alternate donor site; AP, alternate promoter; AT, alternate terminator; ES, exon skip; ME, mutually exclusive exons; RI, retained intron

A total of 10,582 genes had 48,049 events occur in OC.There were seven AS events types in OC,among which ESs is the most common, with 19,251 occurring in 6931 genes, followed by APs and ATs corresponding to 9,689 and 8,453 AS events. The least number was 207 MEs occurring in 201 genes, and the rest were 4006 AAs and 3,497 ADs (Fig. 1a).

### OS-related AS events in OC patients

**Fig. 2:**
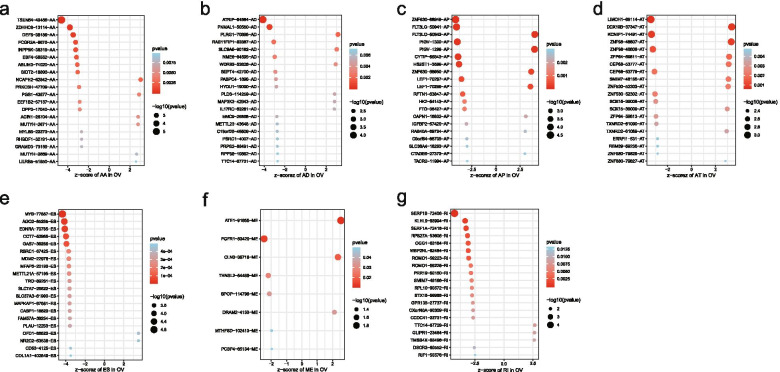
Bubble plots of the alternative splicing events significantly associated with survival in ovarian. The red color and size of the bubble were positively correlated with the survival correlation. There is a corresponding relationship between Z-score and P value in the plots. AA, alternate acceptor site; AD, alternate donor site; AP, alternate promoter; AT, alternate terminator; ES, exon skip; ME, mutually exclusive exons; RI, retained intron

Univariate COX regression analysis of 384 samples showed that there were 31,286 OS related AS events (Additional file 1: Table 1). Volcanic Plot visualizes these AS events (Fig. 1b).Upset plot shows that a gene can have one to seven types of AS events (Fig. 1c).After screening,1524 AS events were significantly correlated with prognosis (Additional file 2: Table 2). In the histogram (Fig. 1d),there are more AS events in each splicing type are conducive to survival than those are bad for survival.The top 20 AS events (8 of ME) in each splicing type are represented by the bubble plots (Fig. 2).

### Establishing OC survival model

**Fig. 3:**
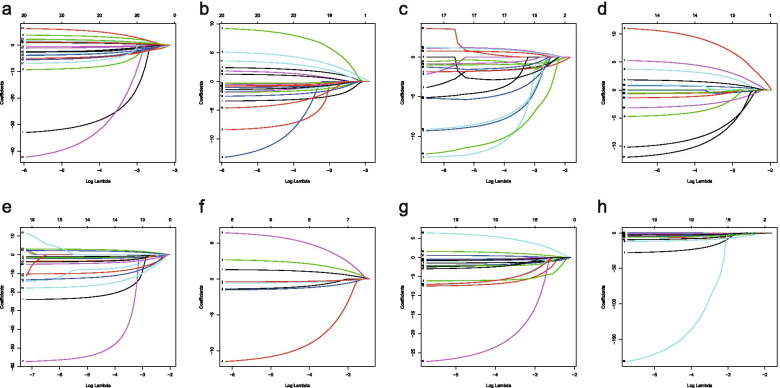
Lasso regression graphs. **a**, Lasso regression of AA; **b**, Lasso regression of AD; **c** ,Lasso regression of AP; **d**, Lasso regression of AT; **e**,Lasso regression of ES; **f**, Lasso regression of ME; **g**, Lasso regression of RI; **h**, Lasso regression of all types .Lasso regression analysis obtained the optimal number of alternative splicing events to construct the survival model. AA, alternate acceptor site; AD, alternate donor site; AP, alternate promoter; AT, alternate terminator; ES, exon skip; ME, mutually exclusive exons; RI, retained intron

**Fig. 4:**
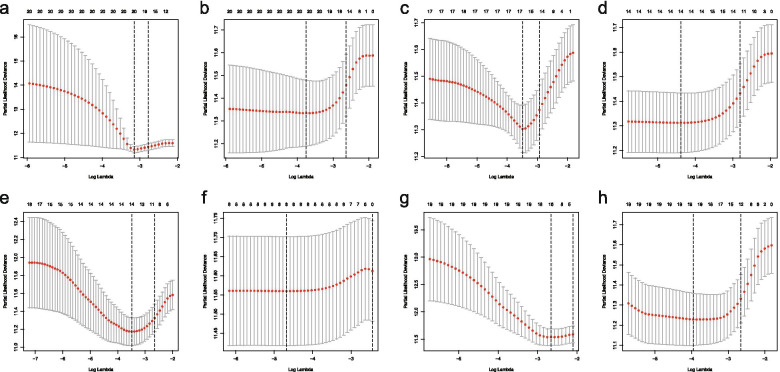
Lasso regression graphs.**a**,Lasso regression of AA;**b**,Lasso regression of AD;**c**,Lasso regression of AP;**d**,Lasso regression of AT;**e**,Lasso regression of ES;**f**,Lasso regression of ME;**g**,Lasso regression of RI;**h**,Lasso regression of all types.Lasso regression was used to obtain the data with the minimum error of the survival model.AA, alternate acceptor site; AD,alternate donor site; AP, alternate promoter; AT, alternate terminator;ES, exon skip; ME, mutually exclusive exons; RI, retained intron

The lambda were selected by Lasso regression (Fig. 3). The adjusted lambda had 17, 20, 14, 20, 14, 8, 16,19AS events corresponding to AP, AA, AT, AD, ES, ME, RI and total splicing events, respectively Fig. 4.

**Fig. 5:**
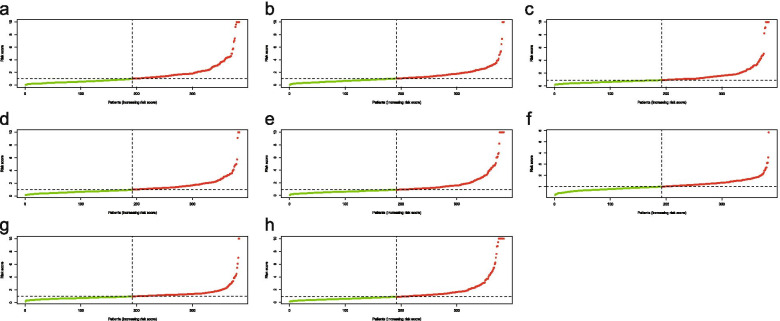
Risk curves of different splicing types in ovarian cancer. **a**, Risk curve of AA; **b**, Risk curve of AD; **c**, Risk curve of AP; **d**, Risk curve of AT; **e**, Risk curve of ES; **f**, Risk curve of ME; **g**, Risk curve of RI; **h**, Risk curve of all types. Green means low risk, red means high risk. AA, alternate acceptor site; AD, alternate donor site; AP, alternate promoter; AT, alternate terminator; ES, exon skip; ME, mutually exclusive exons; RI, retained intron

**Fig. 6:**
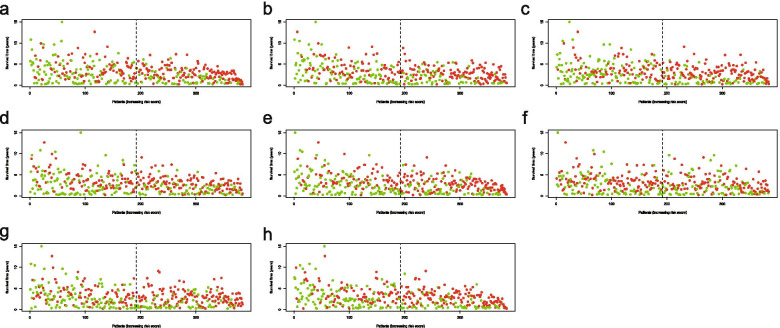
Survival state diagram of ovarian cancer. **a**, Survival state diagram of AA; **b**, Survival state diagram of AD;** c**, Survival state diagram of AP; **d**, Survival state diagram of AT; **e**, Survival state diagrame of ES; **f**, Survival state diagram of ME; **g**, Survival state diagram of RI; **h**, Survival state diagram of all types. The abscissa survival time lengthened gradually, and the green dots represent survival, the red dots represent death. AA, alternate acceptor site; AD, alternate donor site; AP, alternate promoter; AT, alternate terminator; ES, exon skip; ME, mutually exclusive exons; RI, retained intron

**Fig. 7:**
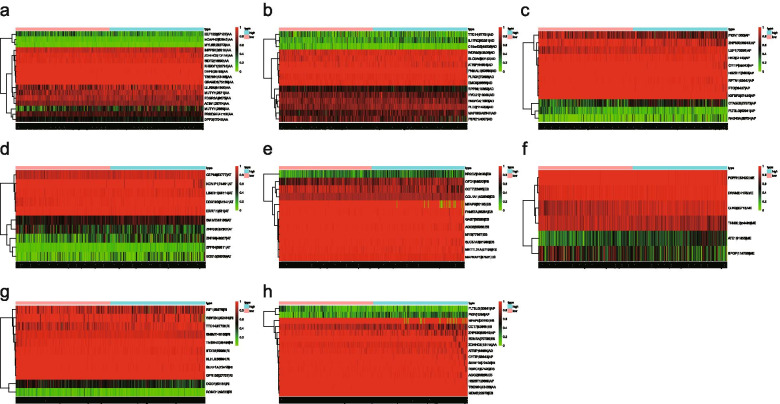
Risk thermography of ovarian cancer. **a**, Risk thermography of AA; **b**, Risk thermography of AD; **c**, Risk thermography of AP; **d**, Risk thermography of AT; **e**, Risk thermography of ES; **f**, Risk thermography of ME; **g**, Risk thermography of RI; **h**, Risk thermography of all types. Risk assessment of alternative splicing events used to construct the survival models. As the risk score increases, the color changes from green to red and then to black to indicate high risk events, and vice versa to indicate low risk events. AA, alternate acceptor site; AD, alternate donor site; AP, alternate promoter; AT, alternate terminator; ES, exon skip; ME, mutually exclusive exons; RI, retained intron

**Fig. 8:**
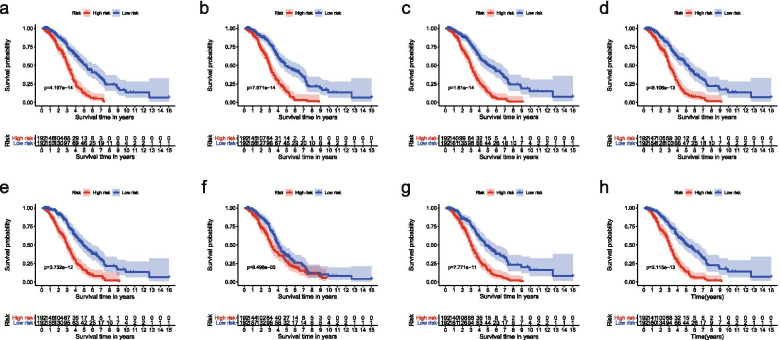
Survival curves of ovarian cancer. **a**, Survival curve of AA; **b**, Survival curve of AD; **c**, Survival curve of AP; **d**, Survival curve of AT; **e**, Survival curve of ES; **f**, Survival curve of ME; **g**, Survival curve of RI; **h**, Survival curve of all types. The survival curves show the differences in survival between the high and low risk groups. AA, alternate acceptor site; AD, alternate donor site; AP, alternate promoter; AT, alternate terminator; ES, exon skip; ME, mutually exclusive exons; RI, retained intron

The Multivariate Cox regression model established eight Cox proportional hazards regression prediction models (Additional files 3, 4, 5, 6, 7, 8, 9, 10, 11, 12, 13, 14, 15 and 16: Tables 3, 4, 5, 6, 7, 8, 9, 10, 11, 12, 13, 14, 15 and 16). The relationship between risk values and prognosis in the high and low risk group was represented by risk score curves,survival status and survival timesplots and PSI value heatmaps (Figs. 5,  6, and 7).The survival curve compared the survival differences between the high-low risk groups. Among the eight models, the difference in AP was the largest and the difference in ME was the smallest (Fig. 8).

**Fig. 9:**
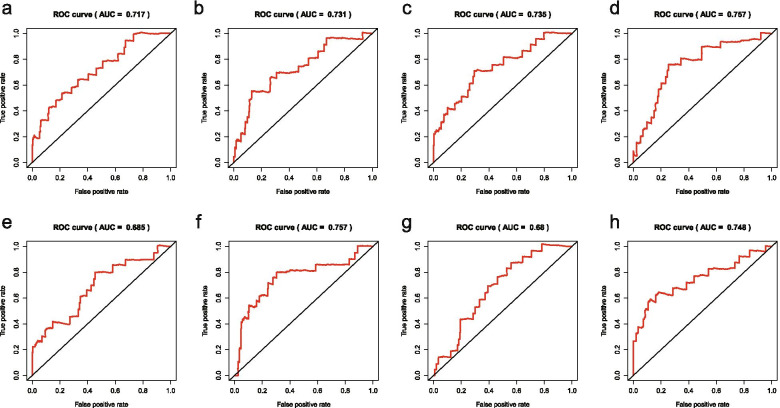
ROC curves of ovarian cancer. **a**, ROC curve of AA; **b**, ROC curve of AD; **c**, ROC curve of AP; **d**, ROC curve of AT; **e**, ROC curve of ES; **f**, ROC curve of ME; **g**, ROC curve of RI; **h**, ROC curve of all types. ROC curves were used to evaluate the accuracy of prognostic models for each type of ovarian cancer. AA, alternate acceptor site; AD, alternate donor site; AP, alternate promoter; AT, alternate terminator; ES, exon skip; ME, mutually exclusive exons; RI, retained intron

ROC curve evaluated 8 prognostic models, the AUC were between 0.680-0.757 (Fig. 9). The AUC of ME and AT were the largest and equal, while the area under the curve of RI and ES were less than 0.70.The evaluation results indicated that the prognostic modes were of great significance in the analysis of the prognosis of OC patients.

### Independent prognostic analysis of OC patients

**Fig. 10:**
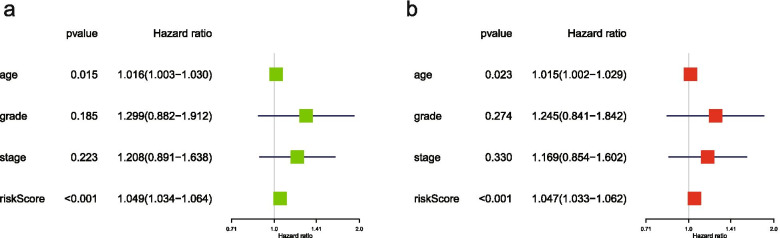
Independent prognostic analysis of ovarian cancer. **a**, single factor independent prognostic analysis of ovarian cancer; **b**, multi-factor independent prognostic analysis of ovarian cancer

Both single-factor independent prognostic analysis and multivariate independent prognostic analysis showed that age and risk score could be used as independent risk factors affecting OS (Fig. 10). In the single-factor independent prognostic analysis (Additional file 17: Table 17), the analysis results of age were P = 0.015, HR = 1.016, and 95%HR CI[1.003-1.030],and the analysis results of risk score were P < 0.001, HR = 1.049, and 95%HR CI[1.034-1.0640].In the multivariate independent prognostic analysis(Additional file 18: Table 18), the analysis results of age were P=0.023,HR=1.015, 95%HR CI[1.002-1.029]) and the analysis results of risk score were P=0.023,HR=1.015, 95%HR CI[1.002-1.029].

### Correlation analysis of AS events and SFs in OC

**Fig. 11:**
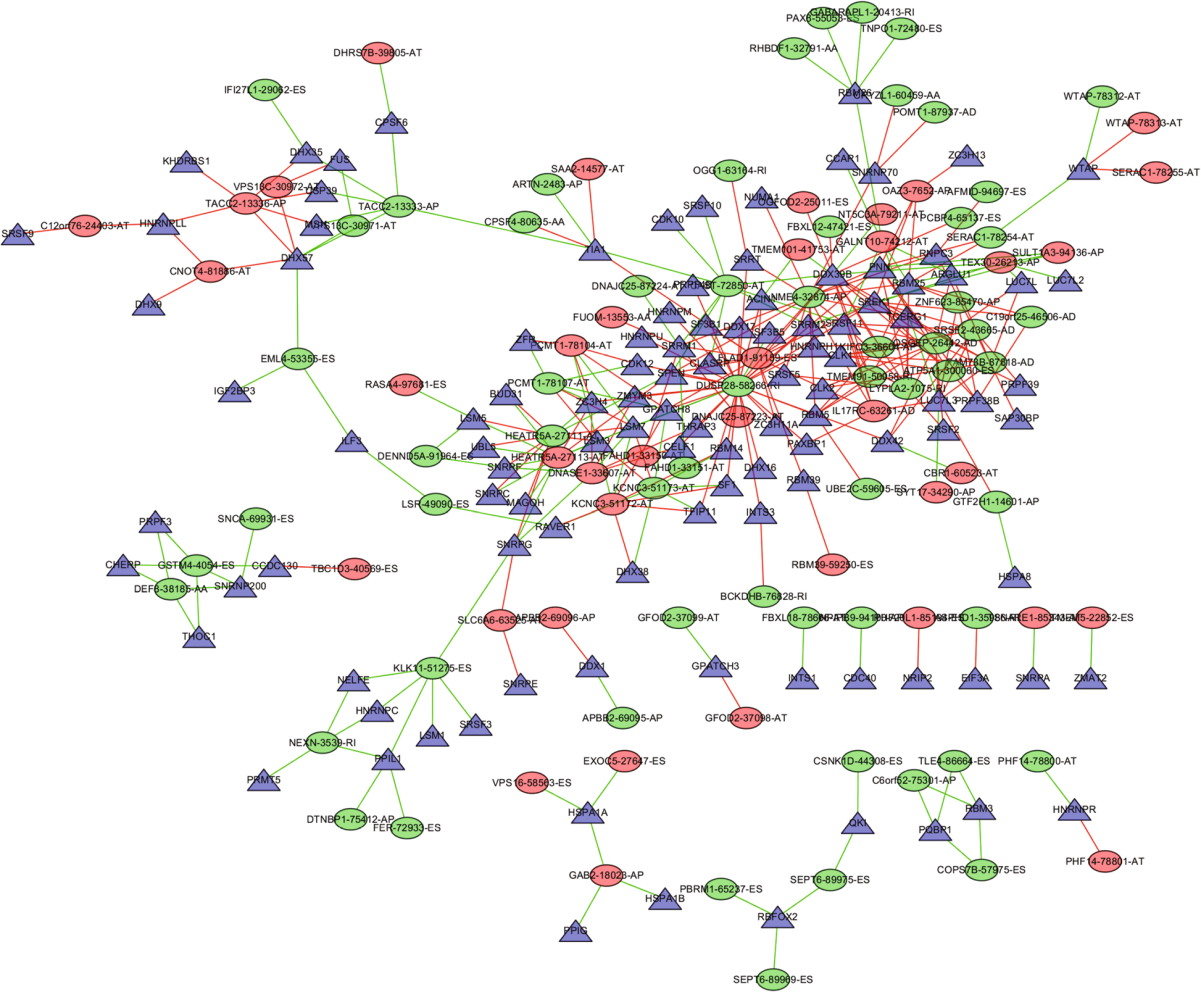
Network of prognostic factors associated with ovarian cancer.Network diagram of the relationship between alternative splicing events and Splicing factors which associated with OC prognosis.The purple triangle represents splicing factors, the red circle represents alternative splicing events that are favorable for survival, the green circle represents alternative splicing events that are unfavorable for survival, the green line represents positive regulation, and the red line represents negative regulation

**Fig. 12:**
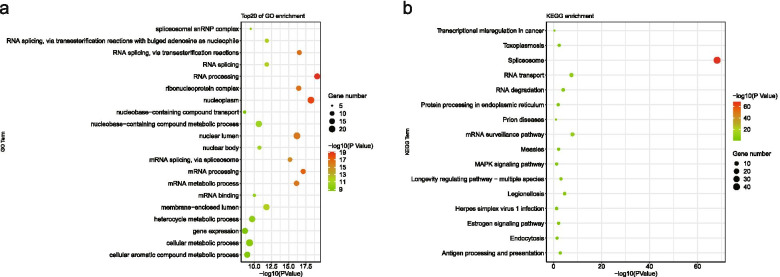
Enrichment analysis of splicing factors. **a**, Gene Ontology enrichment analysis of splicing factors; **b**, Kyoto Encyclopedia of Genes and Genomes enrichment analysis of splicing factors

Univariate COX regression analyzed the correlation between SF genes and AS events.The results showed that 109 SFs were associated with 324 AS events (Additional file 19: Table 19).In the network constructed by Cytoscape (Fig. 11), the relationship between SF genes and AS events are not only one-to-one, but many-to-one or many-to-many.In addition, SFs have positive and negative adjustments for high and low risk events.As shown in the figure , the partial results of GO and KEGG enrichment analysis showed that 109 SFs were involved in a variety of biological processes such as RNA transport, degradation , processing and so on (Fig. 12).

## Discussion

OC is one of the diseases with poor prognosis in gynecological malignancies. In recent years, finding effective prognostic biomarkers for OC has become a hot spot, and it is also an urgent problem to improve the prognosis of OC patients.As is one of the important processes in the regulation of protein diversification. Abnormal regulation of AS can affect human health and also play a role in some cancers.Previous reports have explored the relationship between AS and OC. In this report,the features of the relationship among prognosis, AS and SFs of OC were explored in a more comprehensive way.Besides,some independent prognostic factors of OC were analyzed, providing important basis for systematic analysis.

Prognostic exploration of OC mainly focuses on genomics, in addition to proteomics, immunology, cytokines and other aspects, among which there are complex relationships.For example, the tumor suppressor genes BRCA1 and BRCA2, whose mutations undergo a large number of splicing events, are important components of inherited breast and OC [20–22]. Further investigation revealed that the elimination of BRCA1 mutations through selective mRNA splicing would benefit patients resistant to OC [23]. Recent studies have shown that AAT, NFkB, PMVK and some other proteins are significantly higher in high grade serous ovarian carcinoma than in normal tissue, which can be used as biomarkers for predicting good prognosis [24].Correspondingly, such as PD-1, PD-L1, VAP1, FABP4, and PF4 are associated with poor prognosis [25]; . Interleukin is a series of cytokines produced by many kinds of cells and used a series of cells. There are 38 kinds of interleukin in this family, which can regulate a variety of immune processes.Among them, IL-17 [26, 27], IL-6 [28], IL-8 [28], IL-33 [27], CYR61 [29] and some other cytokines may be involved in the occurrence and development of OC and may be used as prognostic markers of OC.

In this study, we used data from TCGA database, combined with high-throughput sequencing technology and bioinformatics to analyze the relationship between AS, SF and the prognosis of OC.In addition, independent prognostic factors of OC, GO, KEGG analysis of pathways for prognostic related SFs were also explored.Consistent with other studies, our study shows that AS events and SFs play a role in OC, and many splicing events are associated with OC.With the increase of the sample size in this study, the number of AS events in OC was also 48049, with the highest number of ES type [15]. After further treatment, the AS events of TSEN54, MYB, SERF1B, ZNF630 and AGO2 were most closely associated with the prognosis of OC, and they were all good prognostic factors.tRNA splicing endonuclease(TSEN) catalyz tRNA intron removal in eukaryotes,and TSEN54 is a TSEN is one of the core subunits.The gene mutation of TSEN54 mostly leads to nervous system lesions, most studied cause of pontocerebellar hypoplasia.There has been a lack of research on the relationship between OC [30–32]. Previously,MYB is one of the proto-oncogenes, it has become a consensus that there is a relationship between MYB overexpression and poor prognosis of OC. Using the correlation between MYB and OC to evaluate the prognosis and explore potential therapeutic targets has attracted much attention [33–35]. But, the study found that MYB-77867-ES for OC prognosis is favorable AS event. The results suggest that splicing may change the role of genes.AGO2 is a Protein Coding gene, which have effect on cervical cancer, breast cancer and other tumors [36–38]. AGO2 fail in our study it happened happened AGO2-85285-ES is beneficial to the prognosis of OC, but in other studies, AGO2 can have opposite effects on the prognosis of OC through different pathways [39–41]. Few studies have been done on SERF1B and ZNF630,we get SERF1B-72406-RI、ZNF630-88949-AP close contact with OC, they may be effective prognostic biomarkers and therapeutic targets.Among the remaining results, there are many previous studies on CD44 gene.As a non-kinase transmembrane receptor,CD44 can be used as an effective biomarker to predict the prognosis of OC and negatively affect the outcome of OC [42]. CD44 plays a multi-functional role, such as related to cancer stem cells and tumor-associated macrophages, leading to drug resistance of recurrent chemotherapy drugs in OC. It is expected to further dig out effective therapeutic targets to reduce the recurrence rate and drug resistance rate of OC [43, 44]. It interacts with STAT3 to affect a series of processes such as angiogenesis and immune regulation in OC [45–47]. CD44 and STAT3 work together on OC in a variety of way,which can provide various ideas for OC treatment. Consistent with previous researchs, we get CD44-15112-ES can negatively affect the prognosis of OC.

Our carefully designed analysis scheme, it lays the foundation for the feasibility of the prediction models.LASSO regression has unique advantages for survival analysis, which can reduce the complexity of prognostic features, select good model data, and improve the feasibility and accuracy of subsequent survival models [48]. We made full use of the advantages of LASSO regression model in Cox model to further optimize the construction of previous survival models.The survival model was evaluated by ROC curve, and the AUC of the eight models was between 0.68 and 0.757, all of which had good accuracy and feasibility.

Independent prognostic factors are often used as a part of prognostic monitoring and usually divided into clinical features and molecular biology.Common clinical features such as age, performance status, grade, Figo stage, and histology were considered independent prognostic factors for OC [49, 50]. On the other hand, researchers found that low level of AK7, high expression of KIF23, P-gp protein, and SIX-gene signature(TGFBI, SFRP1, COL16A1, THY1, PPIB, BGN) could all be considered independent prognostic factors for OC [51–54]. In this study, we analyzed some clinical traits and found that age can be used as an independent prognostic factor for OC.However, grade and stage were not significant as independent prognostic factors of OC in our study,this may be due to differences in research data and experimental methods.At the same time, we added the score of risk calculated by the prognostic model for evaluation, and risk score was also an independent prognostic factor. This result further demonstrates the strong applicability of the prognostic models, and this design method is relatively new in independent prognostic analysis.

The influence of AS on cancer is beyond question. SF gene indirectly affects the occurrence and development of cancer by affecting AS process [14, 55]. There have been studies on the prognostic relationship between SFs and OC, and the SF we studied came from the data obtained after comprehensive analysis. More SF numbers may lead to more prognostic markers [15]. In this study, 109 SF genes were found to be most relevant for OC prognosis, among which MSI1 is an RNA binding protein that regulates the expression of target mRNAs at the translation level.Initial studies have shown that miR-761 reduces the proliferation and aggressiveness of OC by regulating MSI1 [56]. The increased expression of MSI1 can enhance the malignant characteristics of OC.In addition, MSI1 can increase the expression of p-Bcl-2 by activating the ERK signaling pathway and indirectly increase the drug resistance of chemotherapy drugs [57]. We found that MSI1 can produce positive and negative regulation on different AS events. Incidentally, MSI1 produces positive regulation on negative AS events, while produces negative regulation on positive AS events, and ultimately has negative effects on OC outcomes.RBM3 is the members of the cold shock protein family, it has been shown in a series of studies to be related to the prognosis of breast cancer [58], stomach cancer [59], OC [60], colorectal cancer [61], prostate cancer [62] and other malignant tumors..Asa Ehlen and his team suggest that RBM3 may be an independent prognostic factor for OC, increasing the sensitivity of patients to chemotherapeutic agents and extending the survival of OC patients [63]. Inhibition of Protein Coding gene DDX39B high expression can improve the efficacy of chemotherapy drugs for OC. This is the only study between OC and DDX38B [64]. We found that RBM3 as SF negatively regulates the COPS7B-57975 -ES, and COPS7B- 57975-ES is a good prognostic factors.In other words, RBM3 has a negative prognostic effect on OC.Similarly, the results of this study showed that DDX39b positively affected the prognosis of OC by affecting AS events. This difference may be caused by the mechanism involved in RBM3 and DDX39b that has not been found yet, which needs to be further explored.Among the remaining SFs, the relationship between SFs such as PPIL1, SNRNP200, PQBP1, GPATCH8 and OC have not been explored yet, which can be further studied in the future.

In this paper, the relationship between the prognosis of OC,AS, SF were further explored, but there are still shortcomings.First, the study lacked a control cohort due to the lack of adjacent normal samples around the tumor tissue.Second, the data analyzed in this study came from TCGA and relevant literature, all of which were online data, and the results may not be applicable to clinical patients.Third, the current lack of network data research results applicable to clinical OC patients, the urgent need to establish a clinical cohort to verify the results.Finally, the mechanism of the pathway obtained by GO and KEGG enrichment analysis can be further excavated.

## Conclusions

In conclusion, our results indicated that AS can affect the occurrence and development of OC, and ultimately affect the prognosis of ovarian cancer.On the other hand, SFs can indirectly act on multiple processes of OC development by regulating AS events,and have impact on the survival of OC patients.Further experiments are needed to explore participating pathways to develop effective prognostic biomarkers.

## Supplementary Information

**Additional file 1: Table 1.** Relationship between alternative splicing events and ovarian cancer after univariate Cox regression analysis.HR,hazard ratio,HR>1 are high-risk events, HR<1 are low-risk events.The 95% confidence interval from HR.95L to HR.95H.

**Additional file 2: Table 2.** Alternative splicing events significantly associated with prognosis of ovarian cancer.The P values of all the alternative splicing events were less than 0.05.HR,hazard ratio,HRHR>1 are high-risk events, HR<1 are low-risk events.The 95% confidence interval from HR.95L to HR.95H.

**Additional file 3: Table 3.** Prognostic model of AA events in ovarian cancer.coef is the risk factor.

**Additional file 4: Table 4.** Risk score of AA events in ovarian cancer patients.

**Additional file 5: Table 5.** Prognostic model of AD events in ovarian cancer.coef is the risk factor.

**Additional file 6: Table 6.** Risk score of AD events in ovarian cancer patients.

**Additional file 7: Table 7.** Prognostic model of AP events in ovarian cancer.coef is the risk factor.

**Additional file 8: Table 8.** Risk score of AP events in ovarian cancer patients.

**Additional file 9: Table 9.** Prognostic model of AT events in ovarian cancer.coef is the risk factor.

**Additional file 10:Table 10.** Risk score of AT events in ovarian cancer patients.

**Additional file 11: Table 11.** Prognostic model of ES events in ovarian cancer.coef is the risk factor.

**Additional file 12: Table 12.** Risk score of ES events in ovarian cancer patients.

**Additional file 13: Table 13.** Prognostic model of ME events in ovarian cancer.coef is the risk factor.

**Additional file 14: Table 14.** Risk score of ME events in ovarian cancer patients.

**Additional file 15: Table 15.** Prognostic model of RI events in ovarian cancer.coef is the risk factor.

**Additional file 16: Table 16.** Risk score of RI events in ovarian cancer patients.

**Additional file 17: Table 17.** Single factor independent analysis of ovarian cancer.The 95% confidence interval from HR.95L to HR.95H.P value less than 0.05 indicates that this factor is significant as an independent prognostic factor for ovarian cancer.

**Additional file 18: Table 18.** Multi-factor independent prognostic analysis of ovarian cancer.The 95% confidence interval from HR.95L to HR.95H.P value less than 0.05 indicates that this factor is significant as an independent prognostic factor for ovarian cancer.

**Additional file 19: Table 19.** Analysis of the relationship between splicing factors and variable splicing events.

**Additional file 20: Table 20.** Network node property sheet.

**Additional file 21: Table 21.** Results of splicing factors Gene Ontology enrichment analysis.Input number:The number of splicing factors involved in the pathway.Background number:Number of all genes involved in the pathway.

**Additional file 22: Table 22.** Results of splicing factors Kyoto Encyclopedia of Genes and Genomes enrichment analysis.Input number:The number of splicing factors involved in the pathway.Background number:Number of all genes involved in the pathway.

## Data Availability

Please contact the corresponding author Lihua Yang (13,759,481,789@163.com).

## Abbreviations

OC: Ovarian cancer
AS: Alternative splicing
ROC: Time-dependent receiver operating characteristic
SFs: Splicing factors
OS: Overall survival
AUC: Area under the curve
pre-mRNA: Precursor messenger RNA
HCC: Hepatocellular carcinoma
RNA-seq: RNA sequencing
AA: Alternate acceptor site
AD: Alternate donor site
AP: Alternate promoter
AT: Alternate terminator
ES: Exon skip
ME: Mutually exclusive exons
RI: Retained intronPSI:Percent- splice-in
LASSO: Least absolute shrinkage and selection operator
GO: Gene Ontology
KEGG: Kyoto Encyclopedia of Genes and Genomes
